# Mediating Factors for the Relationship between Stress and Work Ability over Time in Young Adults

**DOI:** 10.3390/ijerph17072530

**Published:** 2020-04-07

**Authors:** Art van Schaaijk, Adnan Noor Baloch, Sara Thomée, Monique Frings-Dresen, Mats Hagberg, Karen Nieuwenhuijsen

**Affiliations:** 1 Amsterdam UMC, University of Amsterdam, Coronel Institute of Occupational Health, Amsterdam Public Health research institute, Meibergdreef 9, P.O. Box 22660, 1100 DE Amsterdam, The Netherlands; m.frings@amsterdamumc.nl (M.F.-D.); k.nieuwenhuijsen@amsterdamumc.nl (K.N.); 2 Biostatistics, School of Public Health and Community Medicine, Institute of Medicine, University of Gothenburg, P.O. Box 463, 405 30 Gothenburg, Sweden; adnan.noor.baloch@gu.se; 3 Department of Psychology, University of Gothenburg, P.O. Box 500, 405 30 Gothenburg, Sweden; sara.thomee@psy.gu.se; 4 Occupational and Environmental Medicine, School of Public Health and Community Medicine, Sahlgrenska Academy and University Hospital, University of Gothenburg, P.O. Box 414, 405 30 Gothenburg, Sweden; mats.hagberg@amm.gu.se

**Keywords:** Mediation, sustainable employability, work ability score, work-private life balance

## Abstract

Stress can affect work ability. The aim of this study was to identify how this pathway is mediated over time in young adults. Participants of the Work Ability in Young Adults cohort were selected. A theoretical framework was built, which lead to a statistical model. Selected dimensions of mediators were recovery, work demands affecting private life, feelings of control over private life, and physical activity in leisure time. A quadruple serial mediation model was built with four mediators. The total effect of stress on work ability was −0.3955, 95% CI [−0.4764, −0.3146]. The total indirect effect amounted to 81% with an effect of −0.3182, 95% CI [−0.3750, −0.2642]. The relationship between stress and Work Ability Score five years later in young adults was mediated by stress five years later, work demands affecting private life, feelings of control over private life and feeling well-rested upon waking. These results indicate that work demands affecting private life and feelings of control over private life are important mediators of the relationship between stress and work ability in young adults. A well-balanced relationship between work and private life can counteract the influence of stress on work ability in this age group.

## 1. Introduction

Work ability is the extent to which people can do their job satisfactorily without doing harm to their mental and physical health and is an important indicator of sustainable employability of workers [1]. Low work ability has been known to be a predictor of absenteeism and early retirement [2,3,4,5]. Stress is one of the most common occupational health problems that influences work ability. In recent years, workloads have increased, which has been accompanied by a growing number of workers reporting severe stress complaints. In Europe, the prevalence of men and women reporting to have work-related stress “always” or “most of the time” is 26% and 27%, respectively [6].

Ilmarinen (2009) stated that young adults need special attention when it comes to work ability. He indicated that it is important to ensure good work ability because at the start of working life workers face high expectations [1]. However, the work ability of young workers is at risk, as it has been reported that this age group has the highest prevalence of stress complaints [6,7]. Young workers are people below the age of 35 [8]. An important feature of young workers is that their working career has just begun. The transition from studies to work in young people may be laden with factors that can affect their health [9]. In this life phase they are often also starting families, for which they need to realize a balance between work and private life. Stress complaints at an early stage of working life can lead to burnout, depression and unfavorable employment outcomes at a later age, according to the life course perspective [10,11]. This life course perspective, an emerging perspective in occupational health, underlines the importance of events during previous life experiences [12]. 

A poor start to working life has indeed been found to have direct and long-term economic, personal, and social consequences [13]. Timely intervention for young workers at risk due to stress may be beneficial for enhancing and maintaining work ability during the rest of their working life [14]. 

Studies in workers at the beginning of their working career with new exposures can contribute to new knowledge in occupational epidemiology [15]. There are indications that—with regard to work stress—early detection and intervention is important when it comes to young people, as work stress precipitates depression and anxiety disorders in young workers [10,16]. Young workers can profit their entire working life from the benefits of early intervention and improve their resilience to workplace stressors. Early intervention may thus promote work participation and prevent permanent exit from the labor market [14]. It has been suggested that interventions may be improved if there is a more in-depth understanding of stress and its pathways [17].

In previous research this relationship between stress and work ability has been well-established in different populations [18,19,20,21,22]. Several models have been developed to study the causes of stress in an occupational setting [23,24]. To determine what happens after stress occurs, and how this affects work ability, it is important that factors affecting this relationship are mapped. It is known that appraised health mediates the effect of stress on work ability in older workers [18]. The mediating effect of health becomes stronger in older workers. However, from the literature it remains unclear what factors mediate the relationship between stress and work ability in young workers. 

The need for a better understanding of the relationship between work ability and mental health underlines the urgency to study the role of factors that influence the relationship between stress and work ability in young workers. As it is hard to eliminate all stress, these factors are important in preventing stress from having a large impact on work ability. 

In Sweden, the prevalence of stress in 2018 was highest among those aged 16–29, with 35% of women and 18% of men in the population indicating feeling stressed [25]. When stress complaints manifest at an early age, the work ability over the course of the working life can be affected as work stress can lead to later in the working life. 

The Swedish Work Ability in Young Adults (WAYA) cohort allows us to study several factors that might influence the relationship between stress and work ability through mediation. In mediation, part of the relationship between stress and work ability can be explained by the presence of another variable/dimension (the mediator). A requirement for mediation is that the mediating factor is related to both stress and work ability. Based on previous research, possible mediating factors present in the WAYA cohort data that are associated with stress and work ability were selected for the current study. These mediating factors can be subdivided into four dimensions: recovery from work, work-home interference, control over private life, and physical activity in leisure time. These four dimensions have all been linked to both stress and work ability, and therefore might mediate this relationship.

The first dimension, recovery from work, can be influenced by stress and the social relationships at work [26]. Stress and the social situation at work are strongly related to disturbed sleep and impaired awakening [27]. In a representative sample of the Swedish working population, 53% of workers reported sleep disorders due to conditions at work [28]. Both stress and work ability are associated with aspects of recovery from work/work-related fatigue and events in private life [29,30]. 

The second dimension, work to private life interference, is mostly caused by job strain and time-interference [31]. This dimension entails the spillover of negative aspects from work to private life. Stress is among others associated with work demands that can interfere with private life [28,32]. This increased negative influence of job demands on private life is associated with reduced work ability in young adults [32].

The third dimension, control over private life, can be affected by (occupational) stress. This entails feeling in control over private life and feeling that one has an influence on desired outcomes i.e., health, interpersonal relations, or financial situations. When stress is high, it can have a negative effect on private life [33]. A lack of control at home is associated with negative health effects, and private life can influence work ability [34,35].

The fourth dimension entails physical activity in leisure time. Physical activity in leisure time differs from physical activity during work [36]. Lower physical activity in leisure time is known to be related to higher stress levels in individuals [37]. A lack of vigorous activity during leisure time is associated with a lower work ability, and the level of physical activity during leisure time is related to work ability [38].

Based on these aforementioned existing relationships in the literature, we expect a mediating effect on the relationship between stress and work ability by the selected dimensions. Intervening on known mediators may help improve sustainable work ability by reducing the negative effect of stress on future work ability. Therefore, results from this study can possibly help occupational professionals monitor work ability in the context of stress and enable work-related interventions. Subsequently, the aim of this study was to map out whether these factors mediate the relationship between stress and work ability over time in younger workers. To enable employers and young workers to eventually maintain sustainable employment during their entire working life, we aim to determine which dimension mediates the relationship between stress and work ability in young adults, and to what extent. Therefore, we drafted the following research question:

*How is the relationship between stress and work ability mediated in young adults over time by recovery, the influence of job demands on private life, feelings of control over private life and physical activity during leisure time?*

## 2. Materials and Methods

### 2.1. Participants and Design

We used questionnaire data from 2012 and 2017 that had been collected in the WAYA cohort of Swedish young adults. The cohort started in 2007 using 20,000 young adults (equally divided between men and women) of 20–24 years old randomly selected from the total population registry held by the Swedish Tax Agency. The first of four questionnaires was sent out in 2007 with follow-up questionnaires in 2008, 2012, and 2017. In these questionnaires, questions on work demands and general health related to these work demands were asked in order to assess occupational health in young adults [39]. The questionnaires were sent out by mail and could be answered by mail or online. Two reminders were sent out per follow-up time-interval. Each questionnaire was accompanied by a lottery ticket worth €1, regardless of whether the questionnaire was filled out and returned or not. During first inspection of respondents, the percentage of young adults working full-time rose from 38% in 2007 to 44%, 67%, and 80% in 2008, 2012 and 2017, respectively. As few young adults had full-time jobs in 2007 and 2008, the time points used for mediation analyses were 2012 and 2017. Persons who answered that they were neither working nor studying (*n* = 250 in 2012 and *n* = 235 in 2017) did not answer the questions on work ability and were excluded from this sample (see Appendix C).

### 2.2. Ethical Declarations

This research was conducted in accordance with the Declaration of Helsinki [40]. The research proposal was submitted to and approved by the Medical Ethical Committee of the Academic Medical Centre and Swedish regional ethical committee Gothenburg Sweden, who decreed that a comprehensive evaluation was not required since this study was not subject to the Medical Research Involving Human Subjects Act (W19_432#19.498; T862-17). The original research proposal was reviewed and approved by the Central Ethical Review Board of the University of Gothenburg, under number T876-11.

### 2.3. Theoretical Framework

When mediation occurs, the relationship between two variables (stress and work ability) can be (partly) explained by another variable (the mediator). A theoretical framework was developed to confirm possible mediating factors. This framework included four possible dimensions of mediators: Recovery, work–home interference, control over private life, and physical activity. In the second part of the analysis, the theoretical framework was expanded with relationships between mediating variables. The mediation pathways were explored based on this theoretical framework.

The mediating variables of interest for these dimensions were as follows: Feeling well-rested upon waking (recovery), demands at work affecting private life (work–home interference), feelings of control over private life (control over private life), and physical activity in leisure time (physical activity). From the theoretical framework, gender was expected to play a role in the pathways of mediation with potentially different relationships between *X* (independent variable, stress), *Y* (dependent variable, work ability) and *M_i_* (possible mediators).

### 2.4. Variables

Work ability was assessed with the Work Ability Score (WAS) i.e., the first question of the Work Ability Index (WAI), where respondents appraised their own work ability on a scale of 0 (no work ability at all) to 10 (best work ability ever experienced). Convergent validity compared to the complete WAI was more than acceptable (*r* = 0.63; *p* < 0.001) [41]. Stress was assessed by a single-item question that was validated against the Maslach burnout inventory (*r* = 0.51; *p* < 0.001) and the mental health subscale of the Short-Form-36 (SF-36) (r = −0.63; *p* < 0.001) and was found satisfactory for measuring stress in various work-life situations [42]. An explanation was included with the question, “Stress means a situation in which a person feels tense, restless, nervous, or anxious, or is unable to sleep at night because his/her mind is troubled all the time. Do you feel this kind of stress these days?”. Response options on this question were recorded on a 5-point scale where a score of 1 corresponded to “not at all” and a score of 5 with “very much”.

The dimension of recovery was assessed by a question on recovery by sleep: “How often in the last 30 days have you woken up feeling well-rested?”. The answer options were “never”, “once/a few times per month”, “several times per week”, and “almost every day”, which were coded as 1 to 4, respectively.

A question on demands at work that negatively affect private life were used for the work–home interference dimension: “Do the requirements of work/studies affect your personal life (leisure, home, and family life) in a negative way?”. There were five answer options were “very rarely”, “quite rarely”, “sometimes”, “quite often”, and “very often”, which were coded as 1 to 5, respectively.

Control over private life was assessed with one question statement: “I feel I have control over and can handle things that happen in my private life”. Respondents could indicate how well this statement matched one of four answer options: “poorly”, “rather poorly”, “well”, or “very well”, which were coded as 1 to 4, respectively.

The physical activity dimension was measured as physical activity during leisure time, and was measured with a single question assessing the average activity level during leisure time in the last twelve months i.e., “How physically active are you and how much do you move in your spare time?”. Four answer options were available, ranging from sedentary leisure time at one extreme (reading, watching TV, computer use or cinema visits) to hard training or competitive sports in competition at the other (running, skiing, biathlon) [43,44,45].

### 2.5. Statistical Analyses

The theoretical model was developed into a mediation model by adding mediators to the statistical model. After the causal steps of Baron and Kenny were confirmed, several models were formed and tested [46]. The first model consisted of one mediating dimension, and the final model consisted of four mediators [47]. The exact way how the statistical model and mediators were formed is based on a new and reliable method, found in Appendix A [48].

This final model leads to a quadruple serial mediation model. The total effect in the quadruple mediation in series is the sum of all pathways in the model and can be written as follows:

*Total effect (c) = Indirect effects + Direct effect (c′)*

*c = a_1_b_1_ + a_2_b_2_ + a_3_b_3_ + a_4_b_4_ + a_1_d_1_b_2_ + a_1_d_4_b_3_ + a_1_d_6_b_4_ + a_2_d_2_b_3_ + a_2_d_5_b_4_ + a_3_d_3_b_4_ + a_1_d_1_d_2_b_3_ + a_1_d_1_d_5_b_4_ + a_1_d_4_d_3_b_4_ + a_2_d_2_d_3_b_4_ + a_1_d_1_d_2_d_3_b_4_ + c′*

This total effect was the sum of all possible pathways leading from *X* to *Y*, direct (*c′*) or via mediating pathways. By adding mediators as regressors, we controlled for the presence of this mediator. These regression analyses were performed for each path, regressing the dependent variable (*Y*) on both the predictor (*X*) and the mediator (*M*). In multiple mediation, the effect of *a_2_* was calculated by regressing *M_2_* on both *X* and *M_1_.* In the final model, the effects were calculated by regressing the outcome (*Y*) on all the connected mediators (*M_1_*, *M_2_*, *M_3_,* and *M_4_*) and the independent variable (*X*). The outcome variable was regressed on all previous predictors to calculate the contribution of each path.

Statistical analyses were performed using IBM SPSS Statistics for Windows, Version 25.0 (IBM Corp, Armonk, NY, USA), in combination with the PROCESS version 3.4 macro by Andrew F. Hayes [49]. Percentages of indirect effects were calculated using Microsoft Office 365 Excel 2016 (Microsoft, Redmont, WA, USA).

## 3. Results

The total number of participants that filled out the questionnaires at 2012 and 2017 was 1733. Of these, 1432 filled out the stress and Work Ability Score on both time-points and were selected for analyses. A flowchart can be found in Appendix C.

The percentages of males in the population was 40% (*n* = 575) and 60% were female (*n* = 857). The means and standard deviations of the WAS at baseline (2012) were 8.2 (±1.7), and five years later these were 7.9 (±1.8). The stress scores of the population at both time points and other baseline population characteristics are shown in Table 1. Descriptive statistics of mediating variables separate for gender in 2012 and 2017 can be found in Appendix D.

All steps of assumptions of mediation according to Baron and Kenny [46] were met for all four dimensions allowing the analysis to proceed to the single mediation step. The relationship between stress and work ability is shown in Table 2.

All single mediation relationships were statistically significant with exception of the indirect effect (*a_1_b_1_*) of physical activity in 2017 (with stress in 2012 as predictor (*X*) and WAS in 2017 as outcome variable (*Y*); For all pathways, see Appendix A). The percentages of the indirect effect (*a_1_b_1_*) of the single mediation model with *X_2012_* on *Y_2017_* mediated by *M_2017_* were 14.0%, 31.9%, 29.1%, and 1.9% for feeling well-rested upon waking, work demands affecting private life, feelings of control over private life, and physical activity in leisure time, respectively. In the model, when stress in 2017 was selected as a mediating variable, the relationship between stress in 2012 and the Work Ability Score in 2017 that went via the indirect pathway (*a_1_b_1_*) was 66% (*p* < 0.001), meaning that there was still a direct effect (*c′*) of stress in 2012 towards work ability in 2017. Stress in 2017 was a strong mediator of the relationship between stress in 2012 and Work Ability Score in 2017.

**Figure A2:**
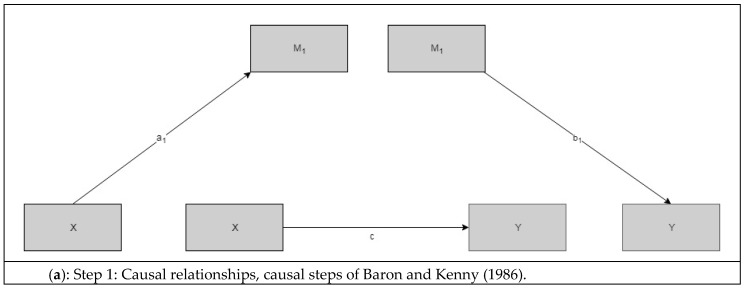

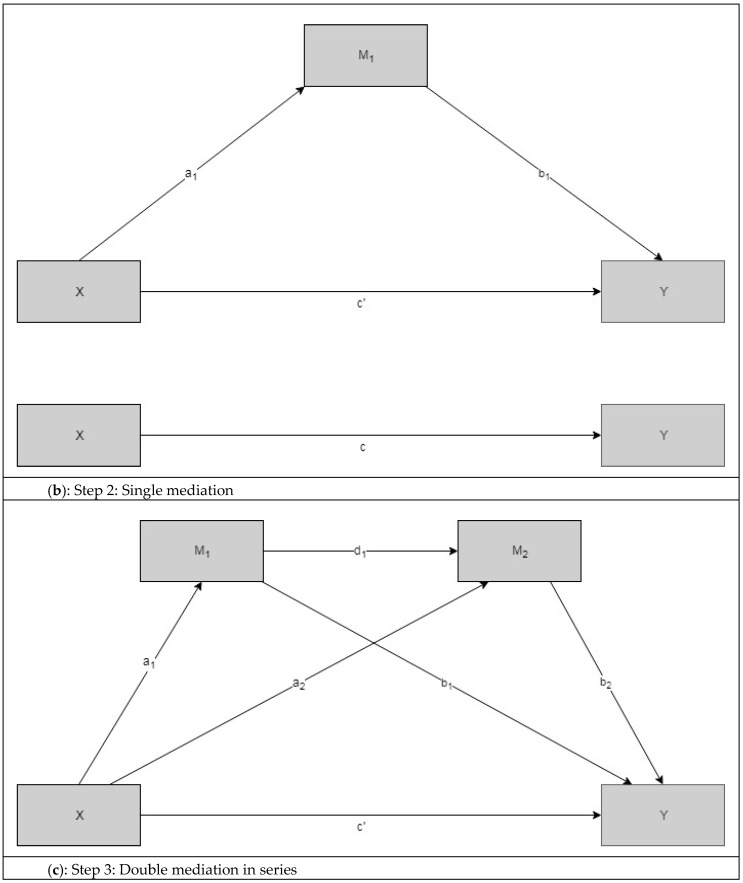

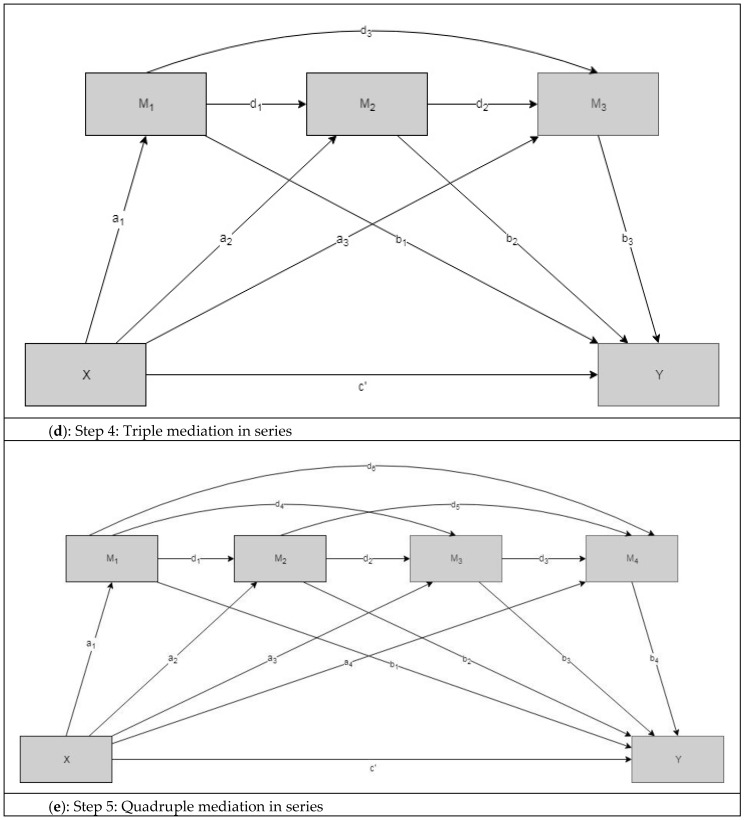

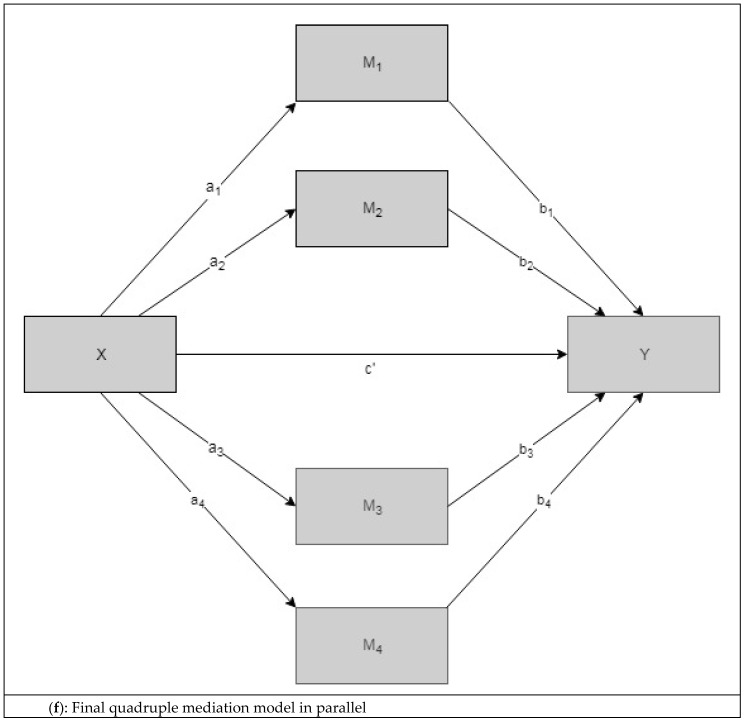
Different mediation steps testing the relationships of the variables.

Starting from the single mediation model, mediators were subsequently added to come to a final model with four mediators (see Appendix A for intermediate steps). Adding pathways through the fourth mediator (*M_4_*) raised the number of indirect effects (*X* to *Y*) from 7 to 15 (see Figure A2d,e). All indirect effects except for indirect effect 14 were statistically significant. The final mediation model consisted of stress in 2017, demands at work affecting private life, feelings of control over private life, and feeling well-rested upon waking. These mediators were connected to each other by paths creating a quadruple serial mediation model. The effects of each pathway are shown in Table 3.

**Figure 1:**
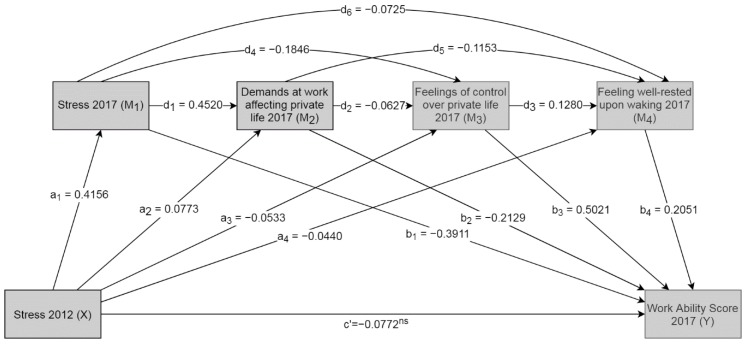
Quadruple mediation with individual effect per path. All paths are significant *p* < 0.05, except c′.

In the final model, the direct effect (*c′*) was negative, but not statistically significant, meaning that the effect of stress in 2012 on work ability in 2017 was almost fully mediated by *M_1_*, *M_2_*, *M_3_*, and *M_4_* (*p* = 0.0570, 95% Confidence Interval (CI) [−0.1568, 0.0023], Degrees of Freedom (DF) (1426)). The selected mediators in the model were feelings of stress, feeling well-rested upon waking, work demands affecting private life, and feelings of control over private life in 2017. All effects for each individual path are shown in Figure 1.

The contribution for each mediator was tested in a parallel format where the *d_i_* paths were eliminated, and the mediating effect only went via *a_i_* and *b_i_* paths through the corresponding mediators. This made it possible to compare the effects of each mediator in the model. The effect which went via pathway *a_1_b_1_* was −0.1626 (95% CI [−0.2068, −0.120]), accumulating to 41%. The effects of *a_2_b_2_*, *a_3_b_3_* and *a_4_b_4_* were −0.0567 (95% CI [−0.0835, −0.0331]), −0.0737 (95% CI [−0.1020, −0.0498]), and −0.0253 (95% CI [−0.0411, −0.0117]), respectively, corresponding to 14%, 19% and 6% of the total effect, indicating that the dimension of feelings of control over private life had the strongest mediating effect. Although *M_3_* had a larger mediating effect than *M_2_*, the theoretical framework suggested that work demands affecting private life preceded feelings of control over private life, resulting in this sequence of mediators in the final serial mediation model.

All four mediators explained 87% of the longitudinal relationship between stress in 2012 and work ability in 2017 for males and 79% for females. In both genders, adding these mediators led to the direct effect (*c′*) not being significant anymore. The mediating effect of *M_1_*, *M_2_*, *M_3_*, and *M_4_* in the quadruple parallel mediation model are displayed in Table 4 for males and Table 5 for females. A slight difference in mediating pathways between both genders becomes clear from Table 4 and Table 5, where the mediating effect of all mediators combined is larger in males. In Appendix E, the serial quadruple mediation model stratified for gender is shown with the effect of each path and pathway.

## 4. Discussion

### 4.1. Findings

The relationship between stress in 2012 and Work Ability Score five years later, i.e., 2017, in young adults was mediated by stress in 2017, work demands affecting private life in 2017, feelings of control over private life in 2017, and feeling well-rested upon waking in 2017, which accounted for 81% of this relationship. This indicates that events at work that affect private life and feelings of control over private life are important for maintaining work ability in young adults with stress complaints. A well-balanced work–private life relationship can counteract the influence of stress on work ability. 

### 4.2. Comparison to Other Studies

Not many studies have been carried out that consider mediators related to work ability. The stress and work ability relationship has been found before, but these studies did not go in-depth regarding other possibly related variables that mediated or moderated this [50]. In our study, the correlation between stress and work ability five years later was found to be moderate and significant, which is in accordance with a previous study that found a trend of long-term stress influencing work ability [51]. Our results show that part of this relationship can be explained by the selected mediating dimensions.

A study by Boström et al. (2012), with the same cohort but at earlier time points, found that job demands that affect private life in a negative way were associated with a reduced work ability [32]. Our study found that this relationship explains part of the stress–work ability relation. This means that part of the relation between stress and work ability can be explained by a spillover of work demands into private life. A study by Geurts et al. (1999) found that negative work–home interference was associated with a decrease in work-related health and general health indicators, which in turn might explain part of the relationship with work ability [52].

Although studies argue that work–home interference can be seen as a cause of stress, our study investigates the influence of demands at work affecting private life and feelings of control over private life on the relationship between stress and work ability [30]. Stress as measured in our study can be caused by other aspects than work–family interference. Geurts et al. (2005) found that higher levels of work–home interference were associated with job pressure, job support, and job control [31]. Peeters et al. (2004) found that job stressors lead to work–home interference and not the other way around [53]. However, two other studies by Dikkers et al. (2006) and Demerouti et al. (2004) found that workload (indicating stress) can be a precursor and consequence of work–home interference [54,55]. Therefore, it still remains unclear how the interaction between stress and work–home interference is constituted.

Yang et al. found that appraised health was a mediator in the stress to work ability relationship for older workers [18]. In a report on psychosocial risks in Europe, it was found that older workers report better work–life balance than young workers. The authors suggested that workers with more experience in the labor market have better working conditions, allowing them to better balance work with other activities [6]. Our study focused on young workers who still need to balance work and private life, showing that the spillover of work into private life and decreased feelings of control over private life should be avoided. Although Geurts et al. (2005) found that home control was not significantly associated with home–work interference, we did see that there was an influence of feelings of control over private life on work ability [31].

### 4.3. Strengths and Limitations

A strength of this study is that we used longitudinal data to study mediation over time in order to make statements about the relationship between stress and work ability over time. Another strength is the depth in which the statistical model was studied. Whereas other studies in this field commonly use two mediators, this study incorporated four mediators while checking the relevance of the serial model. This quadruple mediation model was thoroughly considered by first developing a theoretical framework.

To the best of our knowledge, a study on mediators for the relationship between stress and work ability over time has not been performed before. Therefore, this study adds valuable information regarding prolonged stress and the relationship with work ability.

A limitation of this study is that some of the variables were not validated, as these single item questions represented an entire concept. A consequence can be that the measured concepts do not completely resemble the intended purpose. However, these questions approach the represented dimension as closely as possible. Another possible weakness is that some of the mediator variables were ordinal, but the PROCESS macro uses ordinary least squares regression to handle these variables and predict linearity [56]. All participants were randomly selected from the population to minimize selection bias. Selection bias because of employment status could not have occurred since although persons without a job were invited to participate in the cohort, they did not answer the question on work ability, and were consequently excluded from these analyses. Men were less likely to respond which may put limitations on the generalizability of the findings since there may be participation bias. However, since we conducted an analysis for men and women separately, we have insight in the effect of gender on our findings. A further analysis of people who dropped out between baseline and follow-up showed no differences between the drop-out and continued group, and therefore the risk of attrition bias is low.

### 4.4. Practical Implications

Stress is a common phenomenon in young workers. This study adds information on pathways of how stress can influence work ability in young workers. These pathways can help inform the implementation of interventions in this early phase of a working life. Such interventions may prevent a decreasing trajectory of work ability from regressing [57]. This study adds knowledge about the previously unknown pathways between stress and later work ability in young workers. We now know which mediators for this relationship can be targeted to prevent stress from influencing work ability over time. The results of this study indicate that the relation between stress and work ability is mostly mediated by the spillover of work demands into private life and the feelings of control over private life, indicating that these aspects pose a threat for the sustainable employability of young workers. Therefore, there is an indication that the personal life is affected by stress in the occupational setting, and that the personal life is important for work ability. Policy makers can improve their policies by acknowledging the impact of stress on work ability and their mediating factors. With our results confirming that the private situation is important in the relationship between stress and work ability, it is advisable that both the employer and employee take care of the prevention of and coping with occupational stress, and avoiding work demands to negatively influence personal life.

An important role is therefore placed in boundary management to improve the work–private life balance. Both employer and employee should aim to prevent the work demands affecting private life in a negative way, to prevent declines in work ability over the working life course. Since stress is a common phenomenon in young workers, employees should discuss this issue and possible interventions with their employer at an early stage to prevent decreases in work ability. One such method can be to set boundaries and limits for accessibility outside work hours to aim for a higher feeling of control over their personal life, inhibiting the effect of stress on work ability [39,58,59]. Interventions can be aimed at increasing workplace autonomy and social support at work, which are known to be related to lower work stress levels and less spillover of the negative effect of work demands into private life [60,61].

Early interventions may prevent declines in work ability at later age. When the mediating aspects are managed at the start of and during working life, it can possibly lead to an improved sustainable employment at a later age. An early intervention targeting known mediators can lead to an inhibition of the negative effect of stress on work ability.

Although there were no large differences between men and women, it may be desirable to pay attention to the differences in pathways between men and women when forming strategies to inhibit the effect of stress on work ability.

Implications for further research are that, in the next step, interventions aimed at the mediating factors are tested over time to confirm the preventive efficiency of the interventions to prevent declines in work ability over time. Future research studying work ability needs to take the mediators found in this study into account when mapping out work ability, or when studying work ability in different working populations of all ages.

## 5. Conclusions

The relationship between stress in 2012 and Work Ability Score five years later, i.e., 2017, in young adults was mediated by stress in 2017, work demands affecting private life in 2017, feelings of control over private life in 2017, and feeling well-rested upon waking in 2017, which accounted for 81% of this relationship. Since stress in 2017 mediated a large effect of the longitudinal relationship between stress and work ability, we can conclude that repeated stress is a risk factor for a lower work ability in young adults if not correctly managed.

## Figures and Tables

**Table 1:** Demographic variables mean and standard deviation (SD) of the study population that answered the questions on stress and work ability in 2012 and 2017 (*N* = 1432).

	2012		2017	
	*Mean*	*SD*	*Mean*	*SD*
Age	27.09	1.39	32.09	1.39
Weight (in kg)	72.96	14.53		
Height (in cm)	173.25	9.42		
WAS	8.22	1.67	7.93	1.77
Stress	Not at all	15.2% (*n* = 217)	13.0% (*n* = 186)	
	Just a little	33.0% (*n* = 473)	30.7% (*n* = 440)	
	To some extent	28.3% (*n* = 405)	29.7% (*n* = 426)	
	Pretty much	18.3% (*n* = 262)	19.8% (*n* = 284)	
	Very much	5.2% (*n* = 75)	6.7% (*n* = 96)	
Work, study	Work or internship	73.3% (*n* = 1050)	87.1% (*n* = 1247)	
	Study	12.0% (*n* = 172)	3.9% (*n* = 56)	
	Both	14.7% (*n* = 210)	9.0% (*n* = 129)	
Type of employment	Indefinite contract	68.2% (*n* = 854)	87.9% (*n* = 1208)	
	Probationary period	4.7% (*n* = 59)	2.5% (*n* = 35)	
	Fixed-term contract	9.2% (*n* = 115)	2.7% (*n* = 37)	
	Other fixed-term contract (seasonal or project basis)	17.9% (*n* = 224)	6.8% (*n* = 94)	
Highest completed education	Primary school (9 years)	1.1% (*n* = 16)	1.0% (*n* = 14)	
	High School (12 years)	37.1% (*n* = 531)	24.9% (*n* = 356)	
	University or tertiary, less than 3 years	11.8% (*n* = 169)	11.8% (*n* = 168)	
	University or tertiary, more than 3 years	50.0% (*n* = 715)	62.3% (*n* = 890)	
Family situation	Cohabiting, married, partnership	60.9% (*n* = 869)	75.1% (*n* = 1073)	
	Girlfriend/boyfriend (not living together)	12.0% (*n* = 172)	6.3% (*n* = 90)	
	Single	27.1% (*n* = 387)	18.6% (*n* = 265)	

WAS: Work Ability Score.

**Table 2:** Spearman correlations between stress and work ability variables in the mediation analysis for the years 2012 and 2017 for the total group and separate for males and females (*n* = 1432, *n* = 575, *n* = 857).

		Total	Males	Females
Stress 2012	Stress 2017	0.400	0.388	0.384
	WAS 2012	−0.329	−0.320	−0.322
	WAS 2017	−0.246	−0.231	−0.225
Stress 2017	WAS 2017	−0.429	−0.445	−0.397
WAS 2012	WAS 2017	0.364	0.393	0.337

All correlations were significant (*p* < 0.01).

**Table 3:** Direct and indirect effects with 95% Confidence Inverval (95% CI) for mediating pathways of the relationship between stress in 2012 and work ability in 2017 (*N* = 1432).

	Pathway	Effect	95% CI	% of Total Effect Passed Through Pathway
Total effect	c	−0.3955	[−0.4764, −0.3146]	100
Direct effect	c′	−0.0772 ^ns^	[−0.1568, 0.0023] ^ns^	19.5 ^ns^
Total indirect effect	sum of all below	−0.3182	[−0.3750, −0.2642]	80.5
Indirect effect 1	a_1_b_1_	−0.1626	[−0.2075, −0.1205]	41.1
Indirect effect 2	a_2_b_2_	−0.0165	[−0.0308, −0.0048]	4.2
Indirect effect 3	a_3_b_3_	−0.0268	[−0.0469, −0.0089]	6.8
Indirect effect 4	a_4_b_4_	−0.009	[−0.0195, −0.0006]	2.3
Indirect effect 5	a_1_d_1_b_2_	−0.0402	[−0.0592, −0.0235]	10.2
Indirect effect 6	a_1_d_4_b_3_	−0.0385	[−0.0544, −0.0251]	9.7
Indirect effect 7	a_1_d_6_b_4_	−0.0062	[−0.0123, −0.0017]	1.6
Indirect effect 8	a_2_d_2_b_3_	−0.0024	[−0.0052, −0.0005]	0.6
Indirect effect 9	a_2_d_5_b_4_	−0.0018	[−0.0039, −0.0004]	0.5
Indirect effect 10	a_3_d_3_b_4_	−0.0014	[−0.0031, −0.0003]	0.4
Indirect effect 11	a_1_d_1_d_2_b_3_	−0.0059	[−0.0104, −0.0021]	1.5
Indirect effect 12	a_1_d_1_d_5_b_4_	−0.0045	[−0.0076, −0.0019]	1.1
Indirect effect 13	a_1_d_4_d_3_b_4_	−0.002	[−0.0039, −0.0007]	0.5
Indirect effect 14	a_2_d_2_d_3_b_4_	−0.0001 ^ns^	[−0.0003, 0.0000] ^ns^	0.0 ^ns^
Indirect effect 15	a_1_d_1_d_2_d_3_b_4_	−0.0003	[−0.0007, −0.0001]	0.1

All pathways were significant (*p* < 0.05) with exception of pathways marked with ^ns^ (not significant).

**Table 4:** Direct and indirect effects for mediating pathways for males of the relationship between stress in 2012 and work ability in 2017 in a parallel design (*n* = 575).

	Males		
Pathway	Effect	95% CI	% of Total Effect Passed Through Pathway
Total effect (c)	−0.3825	[−0.5006, −0.2643]	100
Direct effect (c′)	−0.0498 ^ns^	[−0.1604, 0.0608] ^ns^	13.0 ^ns^
Total indirect effect	−0.3326	[−0.4256, −0.2462]	87.0
a_1_b_1_	−0.1809	[−0.2490, −0.1178]	47.3
a_2_b_2_	−0.0505	[−0.0874, −0.0203]	13.2
a_3_b_3_	−0.0855	[−0.1322, −0.0469]	22.4
a_4_b_4_	−0.0158	[−0.0344, −0.0005]	4.1

All pathways were significant (*p* < 0.05) with exception of pathways marked with: ^ns^ (not significant).

**Table 5:** Direct and indirect effects for mediating pathways for females of the relationship between stress in 2012 and work ability in 2017 in a parallel design (*n* = 857).

	Females		
Pathway	Effect	95% CI	% of Total Effect Passed Through Pathway
Total effect (c)	−0.3577	[−0.4677, −0.2477]	100
Direct effect (c′)	−0.0760 ^ns^	[−0.1861, 0.0341] ^ns^	21.2 ^ns^
Total indirect effect	−0.2817	[−0.3547, −0,2154]	78.8
a_1_b_1_	−0.1404	[−0.1991, −0.0839]	39.3
a_2_b_2_	−0.0515	[−0.0874, −0.0213]	14.4
a_3_b_3_	−0.0624	[−0.0989, −0.0320]	17.4
a_4_b_4_	−0.0273	[−0.0500, −0.0089]	7.6

All pathways were significant (*p* < 0.05) with exception of pathways marked with: ^ns^ (not significant).

**Figure A1:**
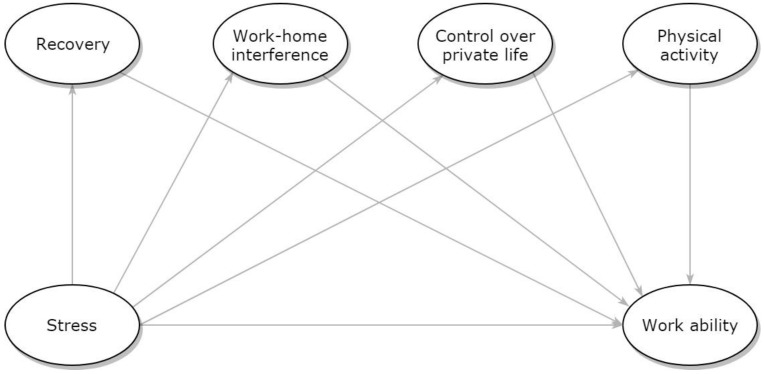
Primary theoretical framework consisting of four dimensions of possible mediating factors of the relationship between stress and work ability.

**Figure A3:**
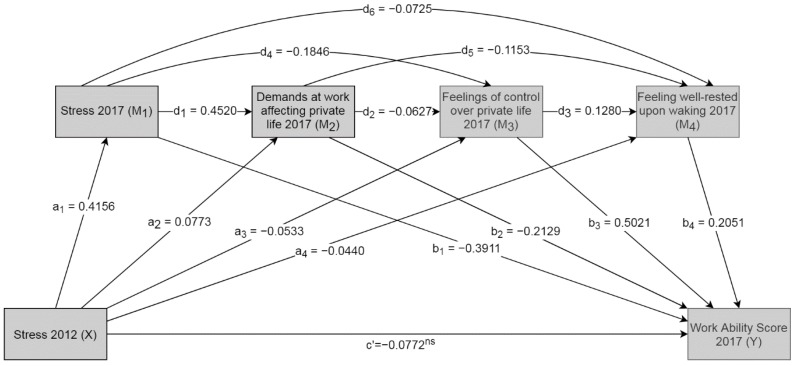
Quadruple mediation with individual effect per path. All paths are significant *p* < 0.05, except c′.

**Figure A4:**
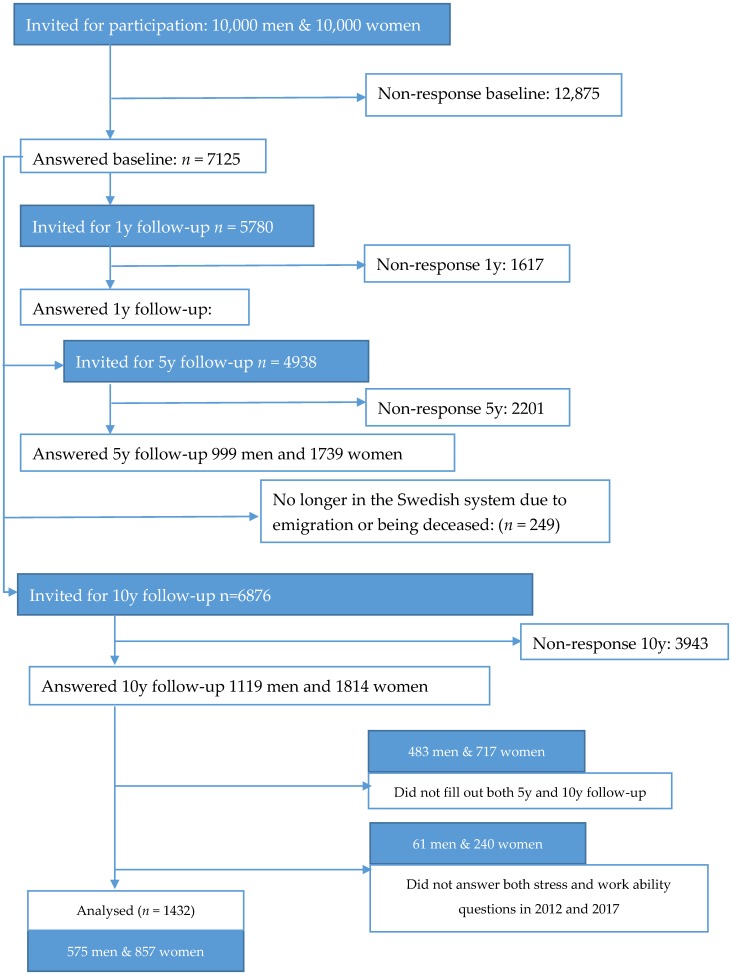
Flowchart of inclusion of the Work Ability in Young Adults (WAYA) cohort.

**Figure A5:**
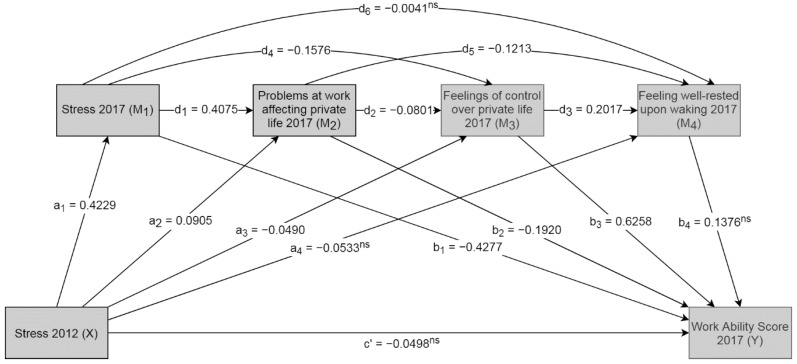
Quadruple mediation with individual effect per path for males. (All paths are significant *p* < 0.05, except a_4_, b_4_, d_6_, and c′).

**Figure A6:**
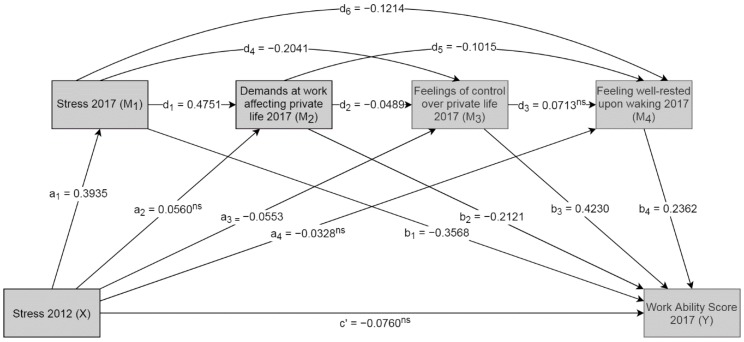
Quadruple mediation with individual effect per path for females. All paths are significant *p* < 0.05, except a_2_, a_4_, d_3_, and c′.

## Appendix A. Methods of Shaping the Statistical Model

Based on the statistical model as shown in Figure A1, a statistical model was built according to the following steps following the criteria for mediation of Baron and Kenny (1986) [46].

First, the requirements for mediation are tested in regression analyses. The assumptions of mediation have to be met to move to the next step. In the first step of our statistical analysis, the requirements for mediation by Baron and Kenny (1986) were tested. The requirements are also known as the causal steps approach [46]. The relationship between *X* and *Y (c)*, *X* and *M (a_1_)*, and *M* and *Y(b_1_)* that are required criteria to determine possible mediation, were studied. The first assumption is that there is a causal relation between the *X* and *Y* variable, where the *X* variable precedes the *Y* variable. In mediation, the *X* variable also needs to precede the mediator variable and a causal relationship needs to be present as well. The second assumption of mediation is that the mediator precedes the Y variable and that there is a causal relationship between these two. In that case, the relationship between *X* and *Y* might be mediated via mediator *M*. The models used in different steps can be seen in Figure A2a.

In the second step, statistical analyses regarding single mediation were performed using the PROCESS version 3.4 macro for IBM SPSS Statistics by Andrew F. Hayes [49]. Every analysis performed using this macro used 10,000 bootstraps for calculation of the indirect effects and the 95% confidence interval. All the significance levels tested were with an α of 0.05. The model used in this macro was model 4 (parallel mediation) [49]. In this step, different models were developed: Cross-sectional models for 2012 and 2017, longitudinal models where the mediator was from 2012 and the outcome was the Work Ability Score in 2017, and another longitudinal model where both the mediator and outcome were from 2017. In this last model, the macro tests for the significance of the mediating effect by calculating the relationship between *X_2012_* on *Y_2017_* that is mediated by *M_2017_*. The effect of *c′* was calculated by dividing the direct effect (*c′*) by the total effect (*c*) to calculate the percentage of direct effect. The percentage of the total effect (*c*) that is explained by the pathway via mediator *M_2017_* was calculated by dividing the indirect effect (*a_1_b_1_*) by the total effect to estimate the percentage of mediation. Where the relationship between *X* and *Y* was smaller when the mediator was present in the model, the direct effect was smaller than the total effect, and where the indirect effect was significant, mediation of the selected mediator was established. This analysis was performed separately for each mediator (feeling well-rested upon waking, work demands affecting private life, feelings of control over private life, and physical activity in leisure time).

After the contribution of each mediator was analyzed, the mediators that were significantly relevant were grouped in a double mediator analysis to form a model with two mediating variables. In this third step, only the mediators that showed a significant indirect effect were selected. Only mediator and outcome variables of 2017 were selected. In this step, all possible combinations of mediators were explored to discover the strongest mediation combination. This multiple mediator model was tested with PROCESS macro with Model 6 (serial mediation).

In the fourth step, the strongest combination of two mediators derived from the third step was added after adding stress in 2017 as the first mediator. The sequence of mediators was based on a theoretical model with interference and causal relationships. The sequence is important because in a serial mediation model, the pathway via *M_1_* can continue via *M_2_*, but not the other way around. Therefore, the dimension that comes first and can affect others, is entered first.

In the fifth and final step of the model building process, the fourth mediator of the theoretical model was added. According to the theoretical model, this mediator was a mediator between the relationship of another mediator and the outcome variable. To test this model, the fourth mediator variable was added in the series. All possible pathways were analyzed and described in the results section.

Following these mediation analyses, the final model was tested with all mediators in parallel to the *X* and *Y* variable to see if pathways explained different parts of mediation. This makes it possible to check if pathways follow multiple mediators in series, or that pathways only go through one mediator (see Figure A2). For the final model, the effect of each pathway (including 95% confidence intervals) were reported, as were the effects of each individual path and percentages for each pathway.

The total effect in the quadruple mediation is series is the sum of all pathways in the model and can be written as follows:

*Total effect (c) = Indirect effects + Direct effect (c′)*

*c = a_1_b_1_ + a_2_b_2_ + a_3_b_3_ + a_4_b_4_ + a_1_d_1_b_2_ + a_1_d_4_b_3_ + a_1_d_6_b_4_ + a_2_d_2_b_3_ + a_2_d_5_b_4_ + a_3_d_3_b_4_ + a_1_d_1_d_2_b_3_ + a_1_d_1_d_5_b_4_ + a_1_d_4_d_3_b_4_ + a_2_d_2_d_3_b_4_ + a_1_d_1_d_2_d_3_b_4_ + c′*

This total effect is the sum of all possible pathways leading from *X* to *Y*, direct (*c′*), or via mediating pathways. The effects of each pathway are calculated by regressing the dependent variable on the independent variable. By adding mediators as regressors, we control for the presence of this mediator. These regression analyses are performed for each pathway, regressing the dependent variable (*Y*) on both the predictor (*X*) and the mediator (*M*). In the multiple mediation model, the effect of *a_1_* is obtained by regressing *M_1_* on *X*, and the effect of *d_1_* is obtained by regressing *M_2_* on *M_1_*. In multiple mediation, the effect of *a_2_* is calculated by regressing both *X* and *M_1_* on *M_2_*. In the final model, the effects are calculated by regressing all the connected mediators (*M_1_*, *M_2_*, *M_3_*, and *M_4_*) and independent variable (*X*) on the outcome (*Y*).

## Appendix B. Results of Steps towards the Statistical Model

*Step 1: Assumptions of Mediation (Relationships between Variables)*

All steps of assumptions of mediation according to Baron and Kenny [46] were met for all four dimensions, except for physical activity in leisure time. We found no evidence for a relationship between stress condition and physical activity in leisure time in 2012, but did find a significant relation in 2017. We can therefore say that the assumptions are met to move to the single mediation step. The relationship between stress and work ability is shown in Table A1.

**Table A1:** Spearman correlations between X and Y variables in the mediation analysis for the years 2012 and 2017 for the total group and separate for males and females (*N* = 1432, *n* = 575, *n* = 857).

		Total	Males	Females
Stress 2012	Stress 2017	0.400	0.388	0.384
	WAS 2012	−0.329	−0.320	−0.322
	WAS 2017	−0.246	−0.231	−0.225
Stress 2017	WAS 2017	−0.429	−0.445	−0.397
WAS 2012	WAS 2017	0.364	0.393	0.337

WAS: Work Ability Score. All correlations were significant (*p* < 0.01).

*Step 2: Single Mediation*

In the second step, 17 single mediation models were tested, (four for each theoretical model and five for the last, as stress in 2017 was included as a mediator): Cross-sectional of 2012, cross-sectional of 2017, longitudinal where only the outcome was from 2017, and longitudinal where both the mediator and the outcome were from 2017. All relationships were statistically significant with exception of the indirect effect (*a_1_b_1_*) of physical activity in 2017 (with stress in 2012 WAS in 2017 as predictor (*X*) and outcome variable (*Y*)). The percentages of the indirect effect (*a_1_b_1_*) of the last theoretical model were 14.0%, 31.9%, 29.1% and 1.9% for feeling well-rested upon waking, work affecting private life, feelings of control over private life, and physical activity in leisure time, respectively.

In the model when stress in 2017 was selected as a mediating variable, the relationship between stress in 2012 and the Work Ability Score in 2017 that went via the indirect pathway (*a_1_b_1_*) was 65.8% (*p* < 0.001), meaning that there was still a direct effect (*c′*) of stress in 2012 towards work ability in 2017. Stress in 2017 was a strong mediator of the relationship between stress in 2012 and Work Ability Score in 2017.

We concluded that, based on the theoretical framework, the stress in 2012 would be maintained as a predictor variable to see whether the relationship between stress in 2012 and future work ability was mediated by the selected mediators of 2017.

*Step 3: Double Mediation*

The third step included mediation of two mediators in series as shown in the Appendix A in Figure A2c. Sixteen models were tested: Stress in 2017 as the first mediator, with all four mediators as second mediator, and the other models consisted of all combinations of the four mediators as first and second mediator (4*3 models).

In these models the pathways between stress in 2012 and the physical activity in leisure time in 2017 were no longer statistically significant. In all models, work demands affecting private life and feelings of control over private life were the largest mediating pathways, with the exception of stress. A model with feelings of control over private life and work demands affecting private life as *M_1_* and *M_2_*, went for 50% via indirect pathways, meaning that the relationship between stress in 2012 and work ability in 2017 is explained by pathways via these two mediators. For the percentage, it does not matter if we use parallel or series, as the effects of *X* on *M_1_* and *M_2_*, and the effects of *M_1_* and *M_2_* on *Y,* are still the same. Only the pathway of *b_1_* goes partly via *d_1_* and *b_2_*. In all models, the *d_1_* pathway was significant, indicating a relationship between both mediators, advocating the parallel design.

*Step 4: Triple Mediation*

In the triple mediation, stress was selected as the first mediating variable (*M_1_*) having the largest mediating effect. In the theoretical framework, stress is seen as a precursor for the other mediator variables. Therefore, stress in 2017 is a precursor for the mediating variables in the remainder of the model. Stress in 2017 is fixed in the models as *M_1_*. In the triple mediation, work demands affecting private life was selected as *M_2_*, and feelings of control over private life was selected as *M_3_*. These two mediators were the largest mediators in the single mediation model, and the combination of these two was the largest in the double mediation model (when stress was not included). In the theoretical model, *M_2_* is preceding *M_3_*, as the feelings of control over private life can be affected by work demands affecting private life. In this model, all direct (*c′*) and indirect (*a_1_b_1_*, *a_2_b_2_*, *a_3_b_3_*, *a_1_d_1_b_2_*, *a_1_d_3_b_3_*, *a_2_d_2_b_3_*, and *a_1_d_1_d_2_b_3_*) pathways as shown in Figure A2d were significant. The total indirect effect was −0.3092 (95% CI [−0.3648, −0.2569]), meaning that with a total effect of −0.3995 (95% CI [−0.4764, −0.3146]), 78% of the effect of stress in 2012 on work ability in 2017 is mediated by *M_1_*, *M_2_*, and *M_3_*.

*Step 5: Quadruple Mediation*

Since sleep quality was related to work ability and stress, this could function as a mediator for the relationship between stress in 2012 and work ability in 2017. However, in the theoretical model sleep quality can be a mediator for the other mediators as well. This means that the relationship between work demands affecting private life and work ability can be mediated by whether or not people felt well-rested upon waking. Therefore, the variable of feeling well-rested upon waking in 2017 is added as last mediator (*M_4_*) to the model. Adding this pathway raised the number of indirect effects from 7 to 15 (see Figure A2e). All indirect effects except for indirect effect 14 were statistically significant. The effects are shown in Table A2.

**Table A2:** Direct and indirect effects for mediating pathways of the relationship between stress in 2012 and work ability in 2017 (*N* = 1432).

	Pathway	Effect	95% CI	% of Total Effect Passed Through Pathway
Total effect	c	−0.3955	[−0.4764, −0.3146]	100
Direct effect	c′	−0.0772 ^ns^	[−0.1568, 0.0023] ^ns^	19.5 ^ns^
Total indirect effect	sum of all below	−0.3182	[−0.3750, −0.2642]	80.5
Indirect effect 1	a_1_b_1_	−0.1626	[−0.2075, −0.1205]	41.1
Indirect effect 2	a_2_b_2_	−0.0165	[−0.0308, −0.0048]	4.2
Indirect effect 3	a_3_b_3_	−0.0268	[−0.0469, −0.0089]	6.8
Indirect effect 4	a_4_b_4_	−0.009	[−0.0195, −0.0006]	2.3
Indirect effect 5	a_1_d_1_b_2_	−0.0402	[−0.0592, −0.0235]	10.2
Indirect effect 6	a_1_d_4_b_3_	−0.0385	[−0.0544, −0.0251]	9.7
Indirect effect 7	a_1_d_6_b_4_	−0.0062	[−0.0123, −0.0017]	1.6
Indirect effect 8	a_2_d_2_b_3_	−0.0024	[−0.0052, −0.0005]	0.6
Indirect effect 9	a_2_d_5_b_4_	−0.0018	[−0.0039, −0.0004]	0.5
Indirect effect 10	a_3_d_3_b_4_	−0.0014	[−0.0031, −0.0003]	0.4
Indirect effect 11	a_1_d_1_d_2_b_3_	−0.0059	[−0.0104, −0.0021]	1.5
Indirect effect 12	a_1_d_1_d_5_b_4_	−0.0045	[−0.0076, −0.0019]	1.1
Indirect effect 13	a_1_d_4_d_3_b_4_	−0.002	[−0.0039, −0.0007]	0.5
Indirect effect 14	a_2_d_2_d_3_b_4_	−0.0001 ^ns^	[−0.0003, 0.0000] ^ns^	0.0 ^ns^
Indirect effect 15	a_1_d_1_d_2_d_3_b_4_	−0.0003	[−0.0007, −0.0001]	0.1

All pathways were significant (*p* < 0.05) with exception of pathways marked with ^ns.^ (not significant).

In the final model, the direct effect (*c′*) was negative, but not statistically significant (*p* = 0.0570, 95% Confidence Interval (CI) [−0.1568, 0.0023], Degrees of Freedom (DF) (1426)), irrespective of the effect of feelings of stress, feeling well-rested upon waking, work demands affecting private life, feelings of control over private life in 2017. All effects for each individual path are shown in Figure A3.

*Step 6: Check Series and Parallel*

The contribution for each mediator was tested in a parallel format where the d-paths were eliminated. This made it possible to compare the effects of each mediator in the model. The effect that went via pathway *a_1_b_1_* was −0.1626 (95% CI [−0.2068, −0.120]), meaning 41.1%. This is the same as in the model with four mediators in series, because all of this effect goes through *M_1_* in both models. For *M_2_*, *M_3_*, and *M_4_*, this percentage changes between both models. In the parallel model the d_i_ paths are not present and the mediating effect of stress in 2012 goes only via *a_i_* and *b_i_* paths through the corresponding mediators. In the parallel model, only the direct mediation of the relationship between stress in 2012 and WAS in 2017 is studied. The effects of *a_2_b_2_*, *a_3_b_3_* and *a_4_b_4_* were −0.0567 (95% CI [−0.0835, −0.0331]), −0.0737 (95% CI [−0.1020, −0.0498]), and −0.0253 (95% CI [−0.0411, −0.0117]), respectively, corresponding to 14.3%, 18.6%, and 6.4% of the total effect. Although *M_3_* has a larger mediating effect than *M_2_*, the theoretical framework suggested that work demands affecting private life preceded feelings of control over private life resulting is this sequence of mediators in the final serial mediation model. In Appendix D, the serial quadruple mediation model stratified for gender is shown.

*Step 7: Mediation Separated for Gender Results*

Analyses separated for gender showed that the effect of the four mediators in parallel design was larger in males than in females. All four mediators explain 87.0% of the longitudinal relationship between stress in 2012 and work ability in 2017 for males, and 78.8% for females. In both genders, adding these mediators lead to the direct effect (*c′*) no longer being significant. The mediating effect of *M_1_*, *M_2_*, *M_3_*, and *M_4_* are displayed in Table A3 for males and A4 for females. A slight difference in mediating pathways between both genders is shown in Table A3 and Table A4.

**Table A3:** Direct and indirect effects for mediating pathways for males of the relationship between stress in 2012 and work ability in 2017 in a parallel design (*n* = 575).

	Males		
Pathway	Effect	95% CI	% of Total Effect Passed Through Pathway
Total effect (c)	−0.3825	[−0.5006, −0.2643]	100
Direct effect (c′)	−0.0498 ^ns^	[−0.1604, 0.0608] ^ns^	13.0 ^ns^
Total indirect effect	−0.3326	[−0.4256, −0.2462]	87.0
a_1_b_1_	−0.1809	[−0.2490, −0.1178]	47.3
a_2_b_2_	−0.0505	[−0.0874, −0.0203]	13.2
a_3_b_3_	−0.0855	[−0.1322, −0.0469]	22.4
a_4_b_4_	−0.0158	[−0.0344, −0.0005]	4.1

All pathways were significant (*p* < 0.05) with exception of pathways marked with: ^ns^.

**Table A4:** Direct and indirect effects for mediating pathways for females of the relationship between stress in 2012 and work ability in 2017 in a parallel design (*n* = 857).

	Females		
Pathway	Effect	95% CI	% of Total Effect Passed Through Pathway
Total effect (c)	−0.3577	[−0.4677, −0.2477]	100
Direct effect (c′)	−0.0760 ^ns^	[−0.1861, 0.0341] ^ns^	21.2 ^ns^
Total indirect effect	−0.2817	[−0.3547, −0.2154]	78.8
a_1_b_1_	−0.1404	[−0.1991, −0.0839]	39.3
a_2_b_2_	−0.0515	[−0.0874, −0.0213]	14.4
a_3_b_3_	−0.0624	[−0.0989, −0.0320]	17.4
a_4_b_4_	−0.0273	[−0.0500, −0.0089]	7.6

All pathways were significant (*p* < 0.05) with exception of pathways marked with: ^ns^.

## Appendix D. Descriptive Statistics of Mediator Variables

**Table A5:** Descriptive statistics of mediator variables for males and females in 2012 and 2017.

	Males (*n* = 575)				Females (*n* = 857)			
	2012		2017		2012		2017	
	%	*n*	%	*n*	%	*n*	%	*n*
**Stress 2012**								
*Not at all*	19.8	114	16.5	95	12.0	103	10.6	91
*Just a little*	36.2	208	35.1	202	30.9	265	27.8	238
*To some extent*	16.1	150	27.0	155	29.8	255	31.6	271
*Pretty much*	14.3	82	15.8	91	21.0	180	22.5	193
*Very much*	3.7	21	5.6	32	6.3	54	6.3	64
Work demands affecting private life in a negative way								
*Very rarely*	30.3	174	20.9	120	17.1	146	13.1	112
*Quite rarely*	20.7	119	26.3	151	19.3	165	21.0	180
*Sometimes*	30.7	176	32.7	188	34.2	292	38.7	332
*Quite often*	14.6	84	16.7	96	22.5	192	19.7	169
*Very often*	3.7	21	3.5	20	7.0	60	7.5	64
Feelings of control over private life								
*Matches very bad*	2.6	15	2.8	16	3.5	30	2.7	23
*Matches pretty bad*	14.1	81	14.6	84	15.9	136	19.1	164
*Matches pretty good*	54.5	312	55.5	319	53.5	547	56.7	486
*Matches very good*	28.8	165	27.1	156	27.1	232	21.5	184
Well-rested upon waking in the last 30 days								
*Never*	8.2	47	10.1	58	7.8	67	14.9	128
*Any/a few times per month*	49.7	285	50.1	288	52.8	452	53.2	456
*Multiple times per week*	32.4	186	29.4	169	29.2	250	25.1	215
*Almost every day*	9.8	56	10.4	60	10.2	87	6.8	58
WAS (0-10)								
*Mean, SD*	8.38	1.54	8.26	1.60	8.11	1.74	7.71	1.84

## Appendix E. Mediation Pathways for Males

**Table A6:** Direct and indirect effects for mediating pathways of the relationship between stress in 2012 and work ability in 2017 in males (*n* = 575).

	Pathway	Effect	95% CI	% of Total Effect Passed Through Pathway
Total effect	c	−0,3825	[−0.5006, −0.2643]	100
Direct effect	c′	−0,0498^ns^	[−0.1604, 0.0608] ^ns^	13.0 ^ns^
Total indirect effect	sum of all below	−0.3326	[−0.4277, −0.2466]	87.0
Indirect effect 1	a_1_b_1_	−0.1809	[−0.2492,−0.1180	43.7
Indirect effect 2	a_2_b_2_	−0.0174	[−0.0399, −0.0014]	4.5
Indirect effect 3	a_3_b_3_	−0.0306 ^ns^	[−0.0705, 0.0043]	8.0 ^ns^
Indirect effect 4	a_4_b_4_	−0.0073 ^ns^	[−0.0208, 0.0029]	1.9 ^ns^
Indirect effect 5	a_1_d_1_b_2_	−0.0331	[−0.0580, −0.0135]	8.7
Indirect effect 6	a_1_d_4_b_3_	−0.0417	[−0.0697, −0.0205]	10.9
Indirect effect 7	a_1_d_6_b_4_	−0.0002 ^ns^	[−0.0055, 0.0048]	0.1 ^ns^
Indirect effect 8	a_2_d_2_b_3_	−0.0045	[−0.0114, −0.0001]	1.2
Indirect effect 9	a_2_d_5_b_4_	−0.0015 ^ns^	[−0.0042, 0.0001]	0.4 ^ns^
Indirect effect 10	a_3_d_3_b_4_	−0.0014 ^ns^	[−0.0045, 0.0003]	0.4 ^ns^
Indirect effect 11	a_1_d_1_d_2_b_3_	−0.0086	[−0.0167, −0.0021]	2.2
Indirect effect 12	a_1_d_1_d_5_b_4_	−0.0029	[−0.0067, −0.0001]	0.8
Indirect effect 13	a_1_d_4_d_3_b_4_	−0.0018 ^ns^	[−0.0047, 0.0000]	0.5 ^ns^
Indirect effect 14	a_2_d_2_d_3_b_4_	−0.0002 ^ns^	[−0.0007, 0.0000]	0.1 ^ns^
Indirect effect 15	a_1_d_1_d_2_d_3_b_4_	−0.0004 ^ns^	[−0.0011, 0.0000]	0.1 ^ns^

All pathways were significant (*p* < 0.05) with exception of pathways marked with ^ns^.

## Appendix F. Mediation Pathways for Females

**Table A7:** Direct and indirect effects for mediating pathways of the relationship between stress in 2012 and work ability in 2017 in females (*n* = 857).

	Pathway	effect	95% CI	% of Total Effect Passed Through Pathway
Total effect	c	−0.3577	[−0.4677, −0.2477]	100
Direct effect	c′	−0.076 ^ns^	[−0.1861, 0.0341]]	21.2 ^ns^
Total indirect effect	sum of all below	−0.2817	[−0.3549, −0.2147]	78.8
Indirect effect 1	a_1_b_1_	−0.1404	[−0.1999, −0.0853]	39.3
Indirect effect 2	a_2_b_2_	−0.0119 ^ns^	[−0.0294, 0.0024]	3.3 ^ns^
Indirect effect 3	a_3_b_3_	−0.0234	[−0.0468, −0.0044]	6.5
Indirect effect 4	a_4_b_4_	−0.0077 ^ns^	[−0.0224, 0.0042]	2.2 ^ns^
Indirect effect 5	a_1_d_1_b_2_	−0.0396	[−0.0672, −0.0159]	11.1
Indirect effect 6	a_1_d_4_b_3_	−0.0340	[−0.0545, −0.0172]	9.5
Indirect effect 7	a_1_d_6_b_4_	−0.0113	[−0.0224, −0.0031]	3.2
Indirect effect 8	a_2_d_2_b_3_	−0.0012 ^ns^	[−0.0036, 0.0004]	0.3 ^ns^
Indirect effect 9	a_2_d_5_b_4_	−0.0013 ^ns^	[−0.0039, 0.0002]	0.4 ^ns^
Indirect effect 10	a_3_d_3_b_4_	−0.0009 ^ns^	[−0.0029, 0.0001]	0.3 ^ns^
Indirect effect 11	a_1_d_1_d_2_b_3_	−0.0039 ^ns^	[−0.0089, 0.0001]	1.1 ^ns^
Indirect effect 12	a_1_d_1_d_5_b_4_	−0.0045	[−0.0090, −0.0012]	1.3
Indirect effect 13	a_1_d_4_d_3_b_4_	−0.0014 ^ns^	[−0.0036, 0.0002]	0.4 ^ns^
Indirect effect 14	a_2_d_2_d_3_b_4_	0.0000 ^ns^	[−0.0002, 0.0000]	0.0 ^ns^
Indirect effect 15	a_1_d_1_d_2_d_3_b_4_	−0.0002 ^ns^	[−0.0005, 0.0000]	0.1 ^ns^

All pathways were significant (*p* < 0.05) with exception of pathways marked with ^ns^.
